# A New Method to Derive Fetal Heart Rate from Maternal Abdominal Electrocardiogram: Monitoring Fetal Heart Rate during Cesarean Section

**DOI:** 10.1371/journal.pone.0117509

**Published:** 2015-02-13

**Authors:** Huei-Ming Yeh, Yi-Chung Chang, Chen Lin, Chien-Hung Yeh, Chien-Nan Lee, Ming-Kwang Shyu, Ming-Hui Hung, Po-Ni Hsiao, Yung-Hung Wang, Yu-Hsin Tseng, Jenho Tsao, Ling-Ping Lai, Lian-Yu Lin, Men-Tzung Lo

**Affiliations:** 1 Department of Anesthesiology, National Taiwan University Hospital, Taipei, Taiwan; 2 Graduate Institute of Communication Engineering, National Taiwan University, Taipei, Taiwan; 3 Research Center for Adaptive Data Analysis & Center for Dynamical Biomarkers and Translational Medicine, National Central University, Taoyuan, Taiwan; 4 Department of Electrical Engineering, National Central University, Taoyuan, Taiwan; 5 Department of Obstetrics and Gynecology, National Taiwan University Hospital, Taipei, Taiwan; 6 Cardiovascular Center, National Taiwan University Hospital, Taipei, Taiwan; Children’s Hospital Boston, UNITED STATES

## Abstract

**Background:**

Monitoring of fetal heart rate (FHR) is important during labor since it is a sensitive marker to obtain significant information about fetal condition. To take immediate response during cesarean section (CS), we noninvasively derive FHR from maternal abdominal ECG.

**Methods:**

We recruited 17 pregnant women delivered by elective cesarean section, with abdominal ECG obtained before and during the entire CS. First, a QRS-template is created by averaging all the maternal ECG heart beats. Then, Hilbert transform was applied to QRS-template to generate the other basis which is orthogonal to the QRS-template. Second, maternal QRS, P and T waves were adaptively subtracted from the composited ECG. Third, Gabor transformation was applied to obtain time-frequency spectrogram of FHR. Heart rate variability (HRV) parameters including standard deviation of normal-to-normal intervals (SDNN), 0V, 1V, 2V derived from symbolic dynamics of HRV and SD1, SD2 derived from Poincareé plot. Three emphasized stages includes: (1) before anesthesia, (2) 5 minutes after anesthesia and (3) 5 minutes before CS delivery.

**Results:**

FHRs were successfully derived from all maternal abdominal ECGs. FHR increased 5 minutes after anesthesia and 5 minutes before delivery. As for HRV parameters, SDNN increased both 5 minutes after anesthesia and 5 minutes before delivery (21.30±9.05 vs. 13.01±6.89, P < 0.001 and 22.88±12.01 vs. 13.01±6.89, P < 0.05). SD1 did not change during anesthesia, while SD2 increased significantly 5 minutes after anesthesia (27.92±12.28 vs. 16.18±10.01, P < 0.001) and both SD2 and 0V percentage increased significantly 5 minutes before delivery (30.54±15.88 vs. 16.18±10.01, P < 0.05; 0.39±0.14 vs. 0.30±0.13, P < 0.05).

**Conclusions:**

We developed a novel method to automatically derive FHR from maternal abdominal ECGs and proved that it is feasible during CS.

## Introduction

Continuous fetal heart rate (FHR) monitoring is a routine for obtaining significant information about the fetal condition during labor. The intrapartum fetal ECG (FECG) has been shown capable of detecting newborn acidemia, and hypoxia [1]. However, the only clinically-available device for FECG analysis requires an invasive fetal scalp electrode, limiting its use to only those with ruptured membranes and a dilated cervix [2]. An alternative method, generally known as the non-invasive FECG, can monitor the FHR through the maternal ECG by placing electrodes on the mother’s abdomen, and many researchers have developed signal processing methods to derive the FECG from the ECG recorded from the mother’s abdomen [3–7]. However, to extract the FHR from the maternal abdominal ECG during cesarean section (CS) remains a challenge since the electrodes cannot be placed properly. The ECG electrodes could only be placed on the lateral sides of the maternal abdomen to avoid interfering with the surgical procedure that can significantly reduce the amplitude of FECG. Moreover, the CS procedure would introduce large motion artifacts and myopotential interference which are difficult to deal with by using traditional methods proposed to extract FECG recorded in the resting state during the prenatal examination. For example, methods based on independent Component Analysis (ICA) might fail since the assumption-the sources (mother and fetal ECGs) are mutually statistically independent-might not be true in the long-term recording for the CS delivery. Also, the frequent position changes of the fetus position during CS delivery can cause the morphological variation of FECG and reconstruction of FECG by the wavelet method with a single selected base (mother wavelet) might be unreliable.

In the present study, we develop a method to derive the FECG by adaptively suppressing the maternal ECG and other interferences from the maternal abdominal ECG. Since the fetal status could not be properly monitored during the CS delivery, it is of clinical importance to monitor FHR during the procedure especially for early identification of fetal distress. Our goal of the present study is to use the novel method to derive FHR noninvasively from the maternal abdominal ECG during CS.

## Materials and Methods

### Ethics Statement

The research was approved by the institutional review board of the National Taiwan University Hospital Ethics Committee (201206049RIC).

### Study subjects and data collection

Subjects scheduled for elective cesarean delivery from September 2012 to December 2012 were included in the study. Subjects admitted for CS were asked if they were willing to join the study when receiving pre-operative anesthesia assessment. Informed consent was obtained one day before in CS after detailed explanation of the study procedure. Only subjects with uncomplicated pregnancies and excluded those with pregnancy-induced hypertension and gestational diabetes were included.

**Fig 1:**
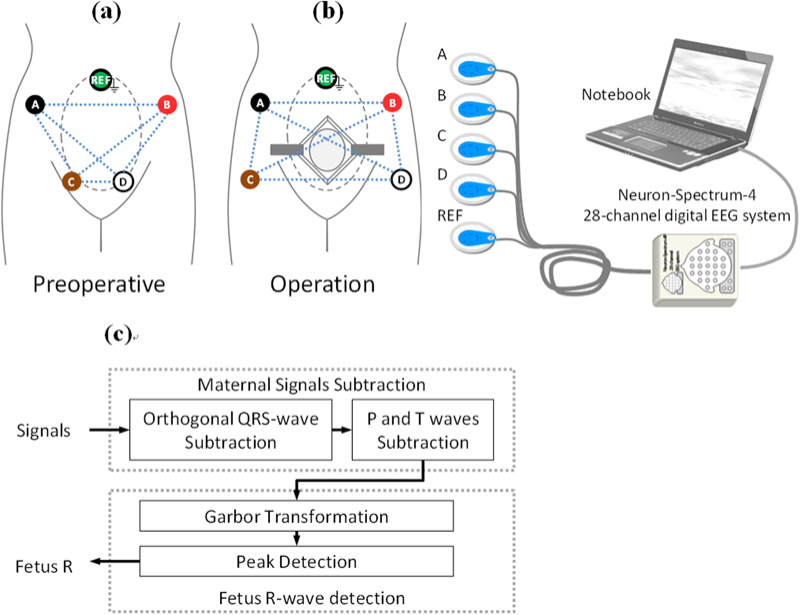
Five abdomen electrodes (four electrodes for signal collecting and one for reference) were placed in different ways for two scenarios. (a) before and (b) during the surgical operations. (c) The work-flow chart of our proposed algorithm. We use four electrodes (marked as A, B, C, and D) for signal acquisition and one (marked as G) for common system reference electrode. Since an voltage signal usually represents a difference between the voltages at two electrodes in EEG measurements, the number of voltage signals in our study would be six (two out of four leads: V_AB_, V_AC_, V_AD_, V_BC_, V_BD_, V_CD_). Noted that the fetal positions are changing during the laboring process, it is our experience that we can ensure the detection of high quality signals (at least one of the voltage signals) by using the applied experimental setting.

After the parturient were transferred into the National Taiwan University Hospital operating room, they were placed in a supine position, equipped with standard monitor, a non-invasive blood pressure cuff, and a pulse oximeter. The ECG signals were obtained from five abdomen electrodes (four electrodes for signal collecting and one for reference) placed away from the surgical site and recorded using a PC-based EEG system (Neuron-Spectrum-4, Neurosoft Company, Russia) in 16 bit, 2 KHz sampling format (see Fig. 1).

After being prehydrated with 1000ml lactated Ringer’s solution, the parturient was turned on her side. A 27-gauge Quincke spinal needle was introduced into the subarachnoid space at the L2–3 or L3–4 interspace in the lateral decubitus position, and 10–12 mg of 0.5% hyperbaric bupivacaine was administered to achieve sensory anesthesia (determined by pin prick) extending to the T4 dematone. Hypotension was treated immediately by intravenous injection of 4–8 mg ephedrine in repeated doses. Other medications were avoided because of their potential influence on measurements. The recordings were performed in the operating room during three different processes: preparing for operation, 5 minutes after spinal anesthesia and 5 minutes before cesarean delivery.

In the study, we included four electrodes with one reference ground. The reference ground was used to serve as a potential reference against which other potentials can be measured and limit the build-up of static electricity, while the other four electrodes were used to do the QRS detection as well as the analysis; since the fetal positions are changing during the laboring process, it is our experience that we can ensure the detection of high quality signals (at least one of the voltage signals) by using the applied experimental setting. After that, we could select the highest quality results for analyzing. The ECGs were recorded during the surgery, and the analyses stated below were performed off-line without any interference to the surgery.

### Maternal QRS-T cancellation

**Fig 2:**
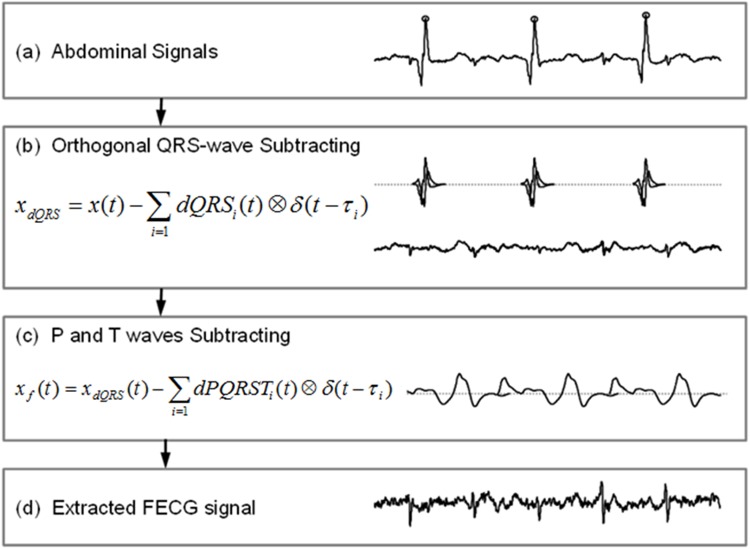
Illustrative maternal of ECG signals processed by the proposed algorithm step by step. (a) An example of raw data with maternal ECG interferences which could be suppressed significantly by the adaptive combination of two orthogonal QRS templates. (b) The maternal P and T waves still appear in the residues even when the QRS complexes are completely removed. (c) The subtraction of P and T waves can be simply implemented by removing the average of all P-T segments.

The FECG is derived from cutaneous electrodes placed on the pregnant women’s abdomen. The FECGs are very weak and usually overwhelmed by the maternal ECG, so the maternal ECG need to be first removed to make way for fetal heart beat detection. Ideally, many studies have proposed to reconstruct the FECG signal by 1) identifying each maternal beat, 2) creating a template acquired from the average of those maternal beats to filter out the blended fetal heart beats and 3) finally subtracting the template from each maternal beat [8, 9]. However, since the waveforms of the maternal ECG are not consistent in different heart beats, this limits effective performance (i.e., the subtraction of the fixed template from an inconsistent maternal ECG will produce a large residue intermittently to severely disturb fetal heart beat detection). An adaptive template therefore was proposed in this study to substantially suppress the material residue. Fig. 2 shows the illustrative maternal ECG signals processed by the proposed algorithm step by step. Briefly, the maternal heart beats were firstly identified and the *i*th maternal QRS wave was denoted as:

$$QRS_{i}(t)=x(t)\cdot rect(\frac{t-\tau_{i}}{W})\;,\;$$ (1)

Where *x*(*t*) is the ECG signals obtained from abdomen electrodes, *τ_i_* is maternal R wave peak time, and *W* is width of the QRS complex. Then a QRS-template, $\overset{\wedge }{QRS(t)}$ was created by averaging the maternal QRS waves with Gaussian weighting function, *G(t)*.

$$\overset{\wedge }{QRS(t)}=\frac{1}{L}\;\sum_{i\;=\;1}^{L}\;\left[QRS_{i}(t)⊗\delta (t+\tau_{i})\right]\;\cdot G(t)\;$$ (2)

Then, Hilbert transform is applied to the resultant QRS-template to generate another basis, $H(\overset{\wedge }{QRS})(t)$ which is mathematically orthogonal to the QRS-template (see lower panel, Fig. 2a) [10, 11].

Respiration and body movements usually cause inconsistent geometrical projection of the depolarization loop onto the electrodes. The adaptive combination of these two orthogonal bases can accommodate the template to the nonstationary change of maternal QRS waveform induced by inconsistent geometrical projection, i.e.,

$$\overset{}{dQRS_{i}(t)}=\;\frac{\int QRS_{i}(t)⊗\delta (t+\tau_{i})\cdot \overset{\wedge }{QRS(t)}}{\int {\left|\overset{\wedge }{QRS(t)}\right|}^{2}}\overset{\wedge }{QRS(t)}+\frac{\int QRS_{i}(t)⊗\delta (t+\tau_{i})\cdot H(\overset{\wedge }{QRS})(t)}{\int {\left|H(\overset{\wedge }{QRS})(t)\right|}^{2}}H(\overset{\wedge }{QRS})(t)$$ (3)

as shown in Fig. 2a, removing such adaptive templates from the ECG recordings can suppress the maternal QRS complex substantially,

$$x_{dQRS}=x(t)-\sum_{i=1}^{}dQRS_{i}(t)⊗\delta (t-\tau_{i})$$ (4)

In some situations, the maternal P and T waves still appear in the residues even after the QRS complex has been perfectly removed (Fig. 2b). Nevertheless, the P and T waves both occupy the low frequency band in the spectrum and are insensitive to body movements. Therefore, the subtraction of the P and T waves can be simply implemented by removing the average of all P-T segments (see Fig. 2b to 2c).

$$\begin{array}{l} x_{f}(t)=x_{dQRS}(t)-\sum_{i=1}^{}dPQRST_{i}(t)⊗\delta (t-\tau_{i})\\ dPQRST_{i}(t)=\frac{1}{L}\sum_{i=1}^{L}\left[x_{dQRS}(t)\cdot rect(\frac{t-\tau_{i}}{W_{PT}})\right]⊗\delta (t+\tau_{i}) \end{array}$$ (5),

where *W*_*PT*_ is the PT interval.

**Fig 3:**
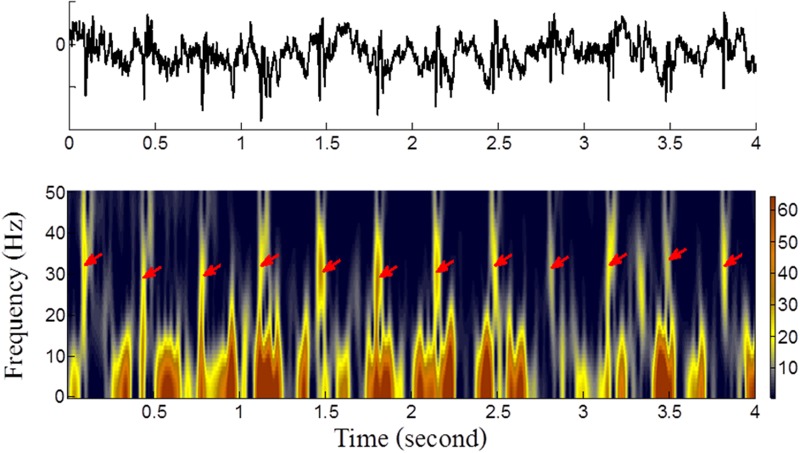
An example of Gabor time frequency representation (lower panel) of ECG signals with suppressed maternal ECG (upper panel). The red arrows indicate the location of the fetal QRS waves, which occupy a frequency range of ~10Hz to 20Hz intermittently that are free of material contamination in Gabor representation.

We apply the Gabor transform to build the time-frequency representation of the reconstructed FECG signals, *x*_*f*_ *(t)* shown in Fig. 3. Apparently, after P-QRS-T cancellation, the fetal heart beats occupy the frequency range of ~10Hz to 20Hz intermittently and exhibit less contamination. Therefore, the fetal QRS waves can be identified by an easy criterion: whether the integrated power within the adaptively selected frequency band crosses the threshold.

The fetal heart beats were detected generally by threshold crossing. To account for variability in waveform amplitude, we set an adaptive threshold on the integrated power within the adaptively selected frequency band. A nonlinear strategy, namely order-statistic filter, was used as the most crucial step to determine the adaptive threshold. The concept of order-statistic filtering was adapted using median filtering algorithm, which is commonly used in imaging denoising [12]. In order-statistic filtering, the input data (i.e., the integration of Gabor power spectrogram within the adaptively selected band) were simply weighted with sliding Tukey window, and then the maximum among the weighted data was obtained as output. In measuring the envelope with order-statistic filtering, the window was shifting 1-point forward each time until the entire set of signals was analyzed, and the procedure was repeated for each windowed data, the fetal heart beat peaks were determined by finding the points with equal magnitude to the integration of Gabor power spectrogram within the adaptively selected band and its envelope. The method basically can reject the pseudo peaks which may be caused by electromyogram or maternal heart beat residue between two consecutive fetal heart beats [13].

To provided evidence to explain the veracity of FECG detection results, we applied our proposed “Fetal QRS Detection” method to the experimental signals download from “Non-Invasive Fetal Electrocardiogram Database” of “PhysioBank” (A valid open-source online database; http://physionet.nlm.nih.gov/pn3/nifecgdb/), and the accurate detection rate is higher than 95%.

### Fetal Heart Rate Variation

**Linear time-domain parameters**. The linear time-domain parameters including mean heart rate, standard deviation of normal-to-normal intervals (SDNN), maximum and minimum heart rate at different stages of the baseline, both 5 minutes after anesthesia, and 5 minutes before CS delivery, were computed. Ectopic beats were inspected visually and rejected by comparison with the adjacent QRS morphologic features. For each stage, the annotated signals of the 90-second ECG recording, which consisted of more than 95% of qualified normal sinus beats, were then used for analysis of HRV. After the outliers of FHR were rejected, we evaluated the mean value of its upper quartile as the mean maximum FHR, and represent the mean value of the lower quartile as the mean minimum FHR.

**Poincaré method to assess the autonomic function**. Unlike the well-established link between the density of the spectrum in specific bands and autonomic system of the adult, the frequency domain analysis of the FHR variation is still too preliminary to show utility. Therefore, instead of frequency domain parameters [14], the Poincaré method was applied to the assessment of the autonomic nervous function [15–17]. Poincaré plot is a simple and easily implemented method which is capable of summarizing an entire RR time series derived from an electrocardiogram in the picture to provide ‘real time’ visualization of data and a quantitative technique which gives information on nonlinear features of autonomic system [18]. Its clinical ability as a predictor of disease and cardiac dysfunction has been proven [19] thus is becoming a popular technique in the field of HRV [20–22]. The current study attempted to apply this method to the assessment of fetal autonomic nervous activities in different stages. The Poincaré plot is a two dimensional scatter distribution, which is constructed by plotting each RR interval against the previous one. Qualitative analysis of the Poincaré plot was proposed by fitting an ellipse to the shape of the plot. The fast beat-to-beat variability (SD1) determined by the dispersion along the minor axis of the ellipse is characterized as a marker of parasympathetic modulation, while the long term beat-to-beat variability (SD2) determined by the dispersion along the major axis of the ellipse is usually characterized as a marker of parasympathetic and sympathetic modulation. The reduced SD1/SD2 ratio may be used as indicative of the increase in the sympathetic modulation.

**Symbolic Dynamics of HRV**. Guzzetti et al. have proposed a nonlinear method of HRV analysis (symbolic dynamic analysis) to quantify the predominance of sympathetic or parasympathetic cardiac modulation in conditions while the use of a linear HRV approach is limited or disputed [23]. The full range of the sequences was divided into 6 levels (from 0 to 5) based on a simple criterion that the 6 levels are equally spaced within the maximum and minimum RR intervals. The symbolic sequences were sorted into categories, and the sequence length L was 3. [23]. All possible patterns were classified into 3 categories: (1) patterns with no variation (0 V; all 3 symbols were at the same level); (2) patterns with 1 variation (1 V; 2 consecutive equal-leveled symbols with one at different level); and (3) patterns with 2 variations (2 V; all symbols were at different levels compared to the previous one). The percentage of the patterns 0 V, 1 V, and 2 V were calculated (see Table 1).

**Table 1:** Temporal evolutional changes of time domain HRV parameters and autonomic nervous function measures (Poincaré method) for Baseline (preparing for operation), 5 minutes after anesthesia and 5 minutes before caesarean-section delivery.

	Baseline	5 minutes after anesthesia	5 minutes before caesarean-section delivery
**Min HR (per minute)**	131.36±6.03	133.75±8.64	142.79±15.71^+^
**Max HR (per minute)**	141.24±7.81	151.16±7.75*	163.68±14.06^++^
**Mean HR (per minute)**	135.88±6.26	141.86±7.69*	153.33±14.51^+^
**SDNN (ms)**	13.01±6.89	21.30±9.05**	22.88±12.01^+^
**SD1**	7.86±4.42	9.75±6.01	9.03±8.06
**SD2**	16.18±10.01	27.92±12.28**	30.54±15.88+
**SD1/SD2**	0.65±0.41	0.38±0.18*	0.33±0.24^+^
**0V**	0.30±0.13	0.37±0.14	0.39±0.14^+^
**1V**	0.37±0.06	0.34±0.05	0.36±0.06
**2V**	0.33±0.10	0.29±0.12	0.25±0.10^+^

Min HR, minimum heart rate; Max HR, maximum heart rate; Mean HR, mean normal-to-normal intervals; SDNN, standard deviation of normal-to-normal intervals; SD1, fast beat-to-beat variability; SD2, the long term beat-to-beat variability; SD1/SD2, the ratio of SD1 to SD2; 0V, patterns with no variation; 1V, patterns with 1 variation; 2V, patterns with 2 variation;

*P < 0.05 baseline vs. 5 minutes after anesthesia;

** P <0.001 baseline vs. 5 minutes after anesthesia;

^+^P <0.05 baseline vs. 5 minutes before caesarean-section delivery.;

^++^P < 0.001 baseline vs. 5 minutes before caesarean-section delivery.

### Statistical Analysis

The average values were expressed as mean ± SD. The normal distribution of the data was first tested using the Shapiro—Wilk test before the subsequent statistical analysis of the computational parameters. The serial data baseline, 5 minutes after anesthesia, and 5 minutes before CS delivery, were then compared by repeated measures analysis of variance. If the result demonstrated a significant time-related effect, either paired Student’s t test or Tukey HSD with Bonferroni correction were performed for the comparisons between groups. The Statistical Package for the Social Sciences (SPSS, version 16.0 for Windows. SPSS Inc., Chicago, II) was used for all statistical analyses. A P value of less than 0.05 was considered statistically significant.

## Results

### Subject characteristics and procedures

A total of 17 parturients scheduled for elective cesarean delivery were included after their written informed consent was obtained. The parturient age was 35.1±3.9 years. The gestational age was 38.7±0.5 weeks. The body weight of the fetus was 3100.8±192.9 g. The Apgar score changed from 8.6±0.7 to 9.8±0.4 1 minute and 5 minutes after delivery respectively. During the study, no complications were noted.

### Temporal evolutional changes of heart rate variability of fetus during delivery

Post hoc pairwise comparisons of HRV parameters against baseline values were shown in Table 1. The mean minimum FHR 5 minutes after anesthesia was similar to the baseline FHR (133.75±8.64 vs. 131.36±6.03 per minute, P > 0.05) but increased significantly before delivery (142.79±15.71 vs. 131.36±6.03 per minute, P < 0.05). As for the mean maximum FHR and the mean FHR, both increased significantly 5 minutes after anesthesia and 5 minutes before delivery as compared with those of the baseline stage. As for the HRV parameters, the SDNN increased both 5 minutes after anesthesia and 5 minutes before delivery (21.30±9.05 vs. 13.01±6.89, P < 0.001 and 22.88±12.01 vs. 13.01±6.89, P < 0.05). The SD1 did not change during anesthesia, while the SD2 increased significantly both 5 minutes after anesthesia (27.92±12.28 vs. 16.18±10.01, P < 0.001) and 5 minutes before delivery (30.54±15.88 vs. 16.18±10.01, P < 0.05) stages. The SD1/SD2 ratio decreased significantly both 5 minutes after anesthesia (0.38±0.18 vs. 0.65±0.41, P < 0.05) and 5 minutes before delivery (0.33±0.24 vs. 0.65±0.41, P < 0.05) stages. For the result of symbolic dynamic analysis, the percentage of 0 V was significantly higher in 5 minutes after anesthesia (0.30±0.13 vs. 0.39±0.14, P < 0.05); on the contrary, the percentage of 2 V was also significantly reduced in 5 minutes after anesthesia (0.33±0.10 vs. 0.25±0.10, P < 0.05); while 0V and 2V percentages did not change during anesthesia.

**Fig 4:**
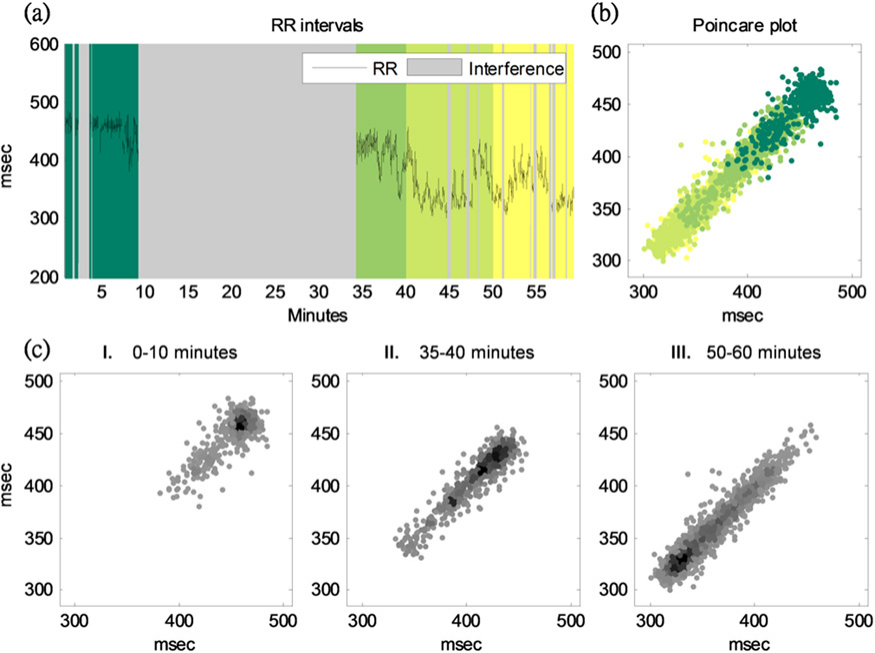
Poincaré plot of the fetal R-R interval time series of a study subject from baseline preparation to the caesarean-section delivery of the fetus. (a) Fetal R-R interval time series of a study subject from baseline preparation to the caesarean-section delivery of the fetus. Three time points-baseline, 5 minutes after anesthesia, and 5 minutes before delivery-were marked by colors of deep green, light green, and yellow. The Poincaré plot for the whole recording is marked with different colors to show the temporal change (b). Detailed Poincaré plot of three stages is provided in Fig. 4c to 4e.

Fig. 4a illustrates the fetal R-R interval time series of a study subject from baseline preparation to the caesarean-section delivery of the fetus. Three time points: baseline, 5 minutes after anesthesia, and 5 minutes before delivery were marked by colors of deep green, light green, and yellow. The corresponding scatter distribution of each RR interval against the previous one for the whole recording are plotted with different colors to show the temporal change (see Fig. 4b). We also provide the detailed Poincaré plot of the three stages in Fig. 4c to 4e. Obviously, both long term beat-to-beat variability (SD2) and mean heart rate showed an increased trend as time evolved.

## Discussion

The Maternal abdominal ECG has been successfully applied to the monitoring of FHR clinically [2–7]. However, to monitor FHR using maternal abdominal ECG during CS is more challenging, since the ECG pads could not be placed in the usual locations leading to low FECG signals. In the current study, we developed a robust method to adaptively subtract maternal ECG and successfully derived FHR from a noisy composite ECG during CS. This method potentially could be applied to monitoring FHR clinically.

A considerable advantage of our method for this composite signal is that, in our algorithm, the maternal QRS template is not fixed but is adaptive. Since the ECG signal is nonstationary and is subject to many sources of interference, our algorithm overcomes the difficulty by generating an orthogonal basis of the QRS template through Hilbert transformation. The adaptive combination of these two orthogonal bases can accommodate the template to the nonstationary change of maternal QRS waveform induced by inconsistent geometrical projection. Through this approach, the maternal ECG could be substantially subtracted in a real-time and adaptive manner. As for the other application of our proposed method, since the atrial activity is uncoupled to ventricular activity during AF, it is also appropriate to apply our proposed method to extract the atrial signal by removing the QRS waveform using the ECGs recorded from the patients who suffered from the atrial fibrillation (AF) [24].

Tulppo et al [18] fitted an ellipse to the shape of the Poincaré plot and defined two standard descriptors of the plot, SD1 and SD2, for quantification of the Poincare plot geometry. These standard descriptors represent the minor axis and the major axis of the ellipse respectively. SD1 (short term variability) is an indirect measure of parasympathetic activity, while SD2 (long term variability) is more strongly related to sympathetic activity than parasympathetic activity [25]. The symbolic dynamic analysis proposed by Guzzetti et al. [23] can be one alternative to quantify the prevalence of sympathetic or parasympathetic cardiac modulation in conditions while the use of a linear HRV approach is limited or disputed. An increase in sympathetic activity results in an increase in the percentage of 0 V [23]. Our results showed that the percentage 0 V was significantly higher 5 minutes before delivery as compared with that in baseline stage. Our study showed that during CS, the SD1 of FHR remained similar while the percentage of SD2 and V0 increased together with increasing FHR, indicating that the sympathetic nerve is activated during anesthesia.

The FHR is controlled by the autonomic nervous system. The inhibitory influence on the heart rate is conveyed by the vagus nerve, whereas excitatory influence is conveyed by the sympathetic nervous system [26]. Stimulations of the peripheral nerves of the fetus by its own activity (such as movement) or by uterine contractions cause acceleration of the FHR [26]. Studies have demonstrated that abnormal FHR during spinal or epidural anesthesia are primarily the results of uterine hypertonus and maternal hypotension [27, 28]. In the current study, the FHR was significantly increased but remained within the normal range, which might be attributed to vasodilatation caused by spinal anesthesia.

There are several potential limitations of the study. First, the study population was small. The effective data are difficult to obtain because of the high possibility of contamination and interference during the delivery; nevertheless, we try recording three additional electrodes simultaneously in order to minimize the weak points. Second, we did not validate our algorithm by invasive fetal scalp ECG. Third, no fetal events occurred during the study. A larger population study could validate the meaning of the derived parameters.

In conclusion, we developed a novel method to automatically derive the FHR from the maternal abdominal ECG and proved that it is feasible in the challenging clinical setting of the cesarean section.
